# A systems perspective on gaps in the person-centered sick leave and rehabilitation process: a Swedish interview study

**DOI:** 10.1080/02813432.2024.2434123

**Published:** 2024-12-02

**Authors:** Märit Löfgren, Karin Törnbom, Daniel Gyllenhammar, Lena Nordeman, Gun Rembeck, Cecilia Björkelund, Irene Svenningsson, Dominique Hange

**Affiliations:** a General Practice / Family Medicine, School of Public Health and Community Medicine, Institute of Medicine, The Sahlgrenska Academy, University of Gothenburg, Gothenburg, Sweden; b Region Västra Götaland, Research, Education, Development & Innovation Primary Health Care, Research, Education, Development & Innovation Center Södra Älvsborg, Sweden; c Department of Social Work, University of Gothenburg, Gothenburg, Sweden; d Department of Clinical Neuroscience, Rehabilitation Medicine, Institute of Neuroscience and Physiology, University of Gothenburg Sahlgrenska Academy, Gothenburg, Sweden; e Department of Technology Management and Economics, Centre of Healthcare Improvements, Chalmers University of Technology, Gothenburg, Sweden; f Institute of Neuroscience and Physiology, Department of Health and Rehabilitation, Unit of Physiotherapy, University of Gothenburg Sahlgrenska Akademin, Gothenburg, Sweden; g Region Västra Götaland, Research, Education, Development & Innovation Primary Health Care, Göteborg, Sweden; h Region Västra Götaland, Research, Education, Development & Innovation Primary Health Care, Research, Education, Development & Innovation Center Fyrbodal, Sweden; i Region Västra Götaland, Research, Education, Development & Innovation Primary Health Care, Research, Education, Development & Innovation Center Skaraborg, Sweden

**Keywords:** Primary health care, sick leave, patient care management, patient-centered care, Sweden

## Abstract

**Background:**

Consensus on priorities to optimize the sick leave and rehabilitation process (SRP) is lacking.

**Objective:**

To explore perspectives of stakeholders in the SRP on bridging the gap between desired process scope, and actual practice, from a multi-professional, multi-organizational, and interdisciplinary approach.

**Design and setting:**

Focus group interviews were conducted with various SRP frontline professionals in Region Västra Götaland, Sweden, using purposive sampling to capture a range of experiences. Participants discussed their perceptions of critical changes and priorities needed to meet patients’ SRP needs in a primary care context. All interviews were analyzed using systematic text condensation, as described by Malterud.

**Subjects:**

General practitioners (*n* = 6), rehabilitation coordinators and licensed healthcare professionals from primary healthcare (*n* = 13), administrators from the Social Insurance Agency, the Employment Agency and Social Services (*n* = 12).

**Results:**

Through data analysis, the following themes emerged: 1) The need for rules and regulations to enable coherent process governance 2) Challenges and opportunities in person-centered SRP: Professional collaboration, organizational priorities, and the need for enhanced leadership, and 3) Balancing resources and patient needs in the SRP: How to improve care quality and accessibility. In summary, participants mainly discussed how to improve process efficiency and quality of care while balancing available resources and a heavy workload. A main goal was to prevent negative spirals of suboptimal decision-making in individual cases, which could lead to increased work, unfortunate outcomes, and patient suffering.

**Conclusions:**

This qualitative study indicated that gaps between a desired process scope and actual practice might be bridged by enabling coherent cross-organizational process governance, prioritizing person-centered ways of working, and balancing available resources and workload. The above changes were believed to improve process quality and overall efficiency.

**Trial registration:**

The study project plan was pre-registered on September 21st, 2020, in the database FOU i VGR (researchweb.org), project number 274941.

## Background

Health-related impaired work ability causes great suffering for individuals. Further, the estimated annual societal costs related to production loss due to sick leave in Sweden amount to SEK 32.6 billion (€ 2.8 billion) for psychiatric illnesses and SEK 38.2 billion (€ 3.2 billion) for physical ailments. A more effective sick leave and rehabilitation process (SRP) should have the potential for better health outcomes, and societal savings [1].

In the Västra Götaland Region, one of the largest in Sweden, primary healthcare centers are staffed by general practitioners (GPs), district nurses, psychologists, psychotherapists, and rehabilitation coordinators [2]. Since 2019, a law was passed requiring primary healthcare providers to include coordination services to support patients on sick leave. The role of rehabilitation coordinators includes coordinating between healthcare, employers, and the Social Insurance Agency to facilitate a smooth return to work. This is part of the Swedish healthcare system’s push for a more person-centered approach to sick leave and rehabilitation. Rehabilitation coordinators have a deep understanding of the SRP and serve as the patient’s primary contact [3]. GPs issue sick leave certificates for medical conditions not requiring specialist care. These certificates are based on assessments of diagnosis, functional and activity limitations, and workability in relation to job requirements [4]. Employers cover the first two weeks of sick leave, after which the Social Insurance Agency takes over, assessing eligibility based on the medical certificate, coordinating vocational rehabilitation, and arranging reconciliation meetings when necessary [4]. Employers remain responsible for adjusting work conditions to support return to work (RTW) and for involving occupational health services as needed [5]. For further information the Swedish sick leave and rehabilitation process, and its stakeholders, are described in Supplementary information 1.

There are extensive reports about the Swedish SRP challenges impairing process quality: inadequate working conditions, and lack of clear routines leading to stress and strain [6, 7], a vague national SRP governance and SRP having low priority in the healthcare system [6], and lack of consensus and clear division of responsibilities between authorities in SRP [8]. Further, previous research in a Swedish context has identified key factors contributing to prolonged sick leave: long waiting times in healthcare [9], the fact that medical diagnoses bring higher social recognition and entitle patients to greater financial benefits than non-medical complaints [10], employers not fulfilling their obligations in SRP [8, 11, 12], general practitioners (GPs) lacking holistic problem understanding in individual SRP cases [12], and medical certificates often lacking adequate information needed for work-oriented rehabilitation [13, 14].

Though multiple SRP challenges are long known, evidence of how to proceed to improve the process needs to be more conclusive. For example, increased collaboration in SRP is generally advocated to enhance the quality of care [11, 13, 15–17], but intervention studies evaluating the effect of the Swedish Social Insurance Agency and primary healthcare collaborating to enable early medical rehabilitation, have shown negative results [18–22], which have been attributed to an "entrapment effect” [19, 22]. Further, early interventions in collaboration with the workplace may positively impact SRP outcome, but knowledge about effective SRP interventions is insufficient. Additionally, recommendations regarding the division of responsibilities among involved primary healthcare professions are unclear, as separate research studies show different SRP professions play essential roles in SRP [23–28].

National investigations, aiming to define the strategic direction of SRP improvement work, have primarily put the emphasis on *either* the social welfare perspective [8], *or* the healthcare perspective [6, 7], resulting in differing recommendations. From the social welfare perspective, increased collaboration between authorities, creating consensus around regaining working capacity being the common SRP goal, digital solutions facilitating information share, strengthening the incentives for employers to support RTW, and the Social Insurance Agency taking greater responsibility for helping patients based on individual SRP needs are underscored [8]. From the healthcare perspective on the other hand, adequate working conditions, a focus shift from describing symptoms of illness to providing practical medical information facilitating the removal of barriers to return to work (RTW) [7], more apparent SRP routines, and increased national process governance are emphasized [6].

SRP routines have been clarified to enhance SRP care, and the importance of patient participation in tailoring activities to individual needs, and care coordination, is highlighted in current SRP guidelines, standards, and acts [29–32].

However, in previous studies we found that the scope of SRP might need to be tuned to optimize SRP outcomes, and that there is a pronounced gap between what is stated in current SRP guidelines, and how the SRP was perceived to work in reality [33, 34]. Further research, and new approaches are needed to understand why SRP change management efforts, to implement improved guidelines and more person-centered ways of working, might miss their targets.

Organization-focused research shows that *quality-driven* change management has the potential to both increase quality and reduce costs [35]. Managing urgent problems, which follow chronic quality problems, may require as much as 50% of the available time [36]. Understanding issues that may arise, and why they arise, and letting target group needs guide improvement initiatives is central to positive outcomes from improvement work [37–39]. On the contrary, carelessly striving to reduce costs in the short perspective often leads to sub-optimizations due to deprived quality, and cost increase, in the long run [35, 40, 41], and improving public organizational service by working solely on a strategic level rarely contributes to a better result, instead the opposite [42]. Further, more than efforts at competence development alone is needed to achieve change when implementing new working methods. Actual change requires adequate organizational prerequisites (including resources), supportive management, and clear priorities [43]. It is also essential that people who need to change their behavior understand why this is important and feel motivated to do so. We need to adopt a respectful understanding that such change can take time; behavioral change requires persistent effort and time, which is unfortunately often overlooked.

As described, there are multiple suggestions on how to improve SRP. Still, consensus on hands-on SRP priorities in practice is lacking, and few studies, or series of studies, have scoped for an ‘above the ground’ perspective involving all of SRP and multiple actor perspectives. There is a need to concretize which SRP actor needs to make precisely which changes, and on which organizational level, to improve SRP as a whole. To enable efficient quality-driven change management, there is a need to understand frontline challenges in SRP from an interdisciplinary research perspective involving both healthcare, public service organizations, and social sciences.

The aim of this study was to examine the perspectives of frontline professionals in the SRP on how to bridge the gap between the desired scope of the process and its actual practice, using a multi-professional, multi-organizational, and interdisciplinary approach.

## Methods

### Study design

This study is part of a broader, comprehensive project aimed at providing decision support for policymakers.

A qualitative study design was employed to explore participants’ thoughts and experiences, focusing on generating professional perspectives and contextual knowledge [37–39]. Participants were selected strategically to provide a comprehensive understanding of the research question from diverse angles [44]. Data collection involved digital and physical focus group discussions, fostering interactions to elucidate participants’ statements and various perceptions [45, 46]. The collected material was extensive and comprised over 12 h of focus group discussions (see data collection) on the SRP process from various perspectives. Our aim was to address three overarching and distinct research questions, which resulted in three articles, of which this is the third. The presentation of study results adheres to the Standards for Reporting Qualitative Research (SRQR) checklist [47].

Authors conducting this research consisted of: ML, a female doctoral student in medicine, GP and M.Sc in Engineering, the second author (KT) is a female PhD and SWR with extensive knowledge in the field of qualitative research, the third author (DG) is a male doctoral student in technology and management with a M.Sc in Engineering, the forth author (LN) is an associate professor and RPT with extensive experience in the SRP process, the fifth author (GR) is a female PhD and RN, RM with experience in both qualitative and quantitative research, the sixth author (CB) is a female GP and senior professor, the seventh author (IS) is a female associate professor and RN with extensive experience in qualitative research, and last author (DH) is a female associate professor and GP.

### Setting and participants

The study involved participants from various organizations participating in the SRP in Sweden, including primary healthcare, the Social Insurance Agency, the Swedish Public Employment Service, and the Social Service. The purposive sampling aimed to capture a broad range of experiences and perspectives of the SRP by considering factors such as organizational affiliation, profession, role in SRP, age, gender, geographical location, experience with and attitude towards SRP. Rehabilitation coordinators from different geographical locations helped recommend participants from their respective parts of the region. A total of 41 frontline professionals were recommended, and after obtaining managerial permissions, 36 were invited to participate, with 31 ultimately comprising the final sample. The participants included social insurance administrators (*n* = 12), GPs (*n* = 6), and rehabilitation coordinators/licensed healthcare professionals (*n* = 13). See Table 1 for description of participants.

**Table 1.** Description of participants (*n* = 31) in a qualitative study exploring perspectives of frontline professionals involved in the sick leave and rehabilitation process (SRP) on how to bridge the gap between the desired process scope, and actual practice.

Focus group	Participants’ organization, profession, or role	Sex (men/women)	Experience from SRP relatively new/experienced	Number of PCCs or municipalities represented	Size of PCC patient population	Geographic representation	Perspectives represented
1	Employment Officers (*n* = 2), Social Insurance Administrators (*n* = 2), Social workers (*n* = 2)	0/6	1/5	3 municipalities	–	Small city (*n* = 4), urban area (*n* = 1), sparsely populated area (*n* = 1)	Work oriented rehabilitation and coordination of SRP cases from the perspective of the Social Insurance Office, the Employment Service, and the Social Services.
2	Employment Officers (*n* = 2), Social Insurance Administrators (*n* = 2), Social workers (*n* = 2)	1/5	2/4	3 municipalities	–	Big city (*n* = 2), small city (*n* = 2), urban area (*n* = 1), sparsely populated area (*n* = 1)	
3	General practitioners (*n* = 4)	1/3	0/4	4 PCCs	4,100-13,000	Big city (*n* = 1), small city (*n* = 2), sparsely populated area (*n* = 1)	General practice, insurance medical responsiblity, primary healthcare local management, local multi-professional teamwork.
4	Rehabilitation coordinators and/or licensed healthcare professionals (*n* = 5)	2/3	0/5	8 PCCs	7,400-18,400	Small city (*n* = 1), urban area (*n* = 3), sparsely populated area (*n* = 1)	Primary healthcare SRP coordinator role, occupational therapy, physiotherapy, psychotherapy, psychology, psychosocial team triage, care manager depression, work oriented rehabilitation, local multi-professional teamwork.
5	Rehabilitation - coordinator role and/or therapist (*n* = 5), General practitioner (*n* = 1)	0/6	2/4	8 PCCs	7,600-10,600	Big city (*n* = 2), small city (*n* = 1), urban area (*n* = 1), sparsely populated area (*n* = 2)	These focus groups captured mixed perspectives from various roles, professions and different SRP stakeholders, as the rehabilitation coordinators were either licensed healthcare professionals or had prior experience working with other SRP stakeholders.
6	Rehabilitation - coordinator role and/or therapists (*n* = 3), General practitioner (*n* = 1)	1/3	2/2	4 PCCs	9,200-11,700	Big city (*n* = 1), small city (*n* = 2), sparsely populated area (*n* = 1)	

The study adopts a multi-professional, multi-organizational, and interdisciplinary approach within a primary healthcare context. The participants were sourced from primary healthcare, the Swedish Social Insurance Agency, the Swedish Public Employment Service, and the Social services. They represented diverse professions and perspectives, with most having considerable experience (>5 years) within the SRP, while some were relatively new in the field. They came from different primary care centers (PCCs) and municipalities across the region Västra Götaland, Sweden, encompassing both small and large units as well as urban and sparsely populated areas.

There are organizational nomenclature variations within SRP, but we will henceforth refer to SRP users as ”patients” for clarity. Further, although patients and employers are stakeholders in the SRP, we will use “stakeholders” to specifically refer to the SRP organizations discussed in this article: primary healthcare, the Social Insurance Agency, the Swedish Public Employment Service, and Social Services. We will also use the term ‘professionals’ to refer to individuals with specific expertise who are acting within their professional capacity, and as frontline professionals in direct contact with patients.

### Data collection

Four digital and two physical focus group discussions were conducted between September and October 2021, each lasting two hours and comprising 4-6 participants. There were both focus groups where a single profession/role shared their specific experiences, as well as mixed groups. The aim in composing the groups was to balance unfiltered insights from each profession’s perspective with the opportunity to bring together different viewpoints. For details on group composition, see Table 1. The physical group discussions took place on the university premises. A moderator and an observer facilitated the discussions. Participants discussed their SRP experiences from their professional roles, guided by a semi-structured interview guide (see Appendix I, supplementary material). Open-ended and probing questions were employed to explore SRP-related experiences comprehensively. All predefined research questions were discussed in each focus group: SRP outcomes, work methodologies, overall service experiences, organizational support, and potential process improvements. Field notes were discussed after each focus group to optimize the continued data collection.

### Data analysis

The focus groups were audio-recorded, transcribed, and pseudonymized to ensure anonymity. Data was analyzed employing systematic text condensation, following Malterud’s approach [48]. After reading the interviews, two researchers (ML and DG) independently identified themes, reaching a consensus on preliminary themes for the continued analysis. Meaning units were then identified by the two researchers individually, and grouped according to the preliminary themes using the NVivo program [49]. During the analysis, it became evident that the management scholar, and the primary healthcare scholar, approached data from different perspectives depending on their respective research fields: a ”Social Welfare System” perspective, and an ”Optimizing patients’ sustainable health” perspective. Two sets of codes with thematic sub-codes were worked out to enable focus while maintaining the interdisciplinary approach. DG and ML after that performed the analysis as separate processes, though they participated in each other’s processes, giving feedback on interpretations, and analysis. A management-oriented article was published in 2023 [50].

The content of the meaning units, that had been sorted into code groups and sub-codes to explore the predefined research questions, was condensed. Quotes were integrated into the final step to refine and contextualize the condensates into a coherent analytic text.

### Reflexivity

The interviewers, ML and DG, discussed their preconceptions before conducting the focus groups. This aimed to enhance reflexivity and serve as a reminder to avoid inadvertently influencing the participants during the interviews. These discussions persisted throughout the process to ensure the analysis remained faithful to the collected data. All data underwent thorough deliberation among the authors, carefully examining ambiguities until a consensus was achieved.

## Results

In the analysis of interview data, three codes related to improving SRP by enabling a person-centered and cohesive process emerged: 1) The need for rules and regulations to enable coherent process governance, 2) Challenges and opportunities in person-centered SRP: Professional collaboration, organizational priorities, and the need for enhanced leadership, and 3) Balancing resources and patient needs in the SRP: How to improve care quality and accessibility. See Table 2 for analysis structure and summary.

**Table 2.** Analysis structure and summary of professionals’ perspectives on how to bridge the gap between the desired sick leave and rehabilitation process (SRP), and actual practice, in a primary care context.

Aim	Code groups	Subgroups	Condensed findings	Implications on SRP improvement strategies
To examine the perspectives of professionals in the SRP on how to bridge the gap between the desired process scope, and actual practice, from a multi-professional, multi-organizational, and interdisciplinary approach.	The need for rules and regulations to enable coherent process governance	Goals and definitions Governance strategy Organizational culture and mentality The dual role of the employer	Existing rules and regulations were perceived to need reworking with the aim to allow coherent cross-organizational process governance to prevent fragmentation, confusion, and ‘not-my-problem’ mentality.	This qualitative study indicates that gaps between desired process scope and actual practice may be bridged by enabling coherent cross-organizational process governance, prioritizing person-centered ways of working, and balancing available resources and workload. The above changes were believed to enable both improved process quality and overall efficiency.
	Challenges and opportunities in person-centered SRP: Professional collaboration, organizational priorities, and the need for enhanced leadership	Making use of the expertise of different professions Overall liability Continuity of relations	Person-centered organizational priorities, to enable an SRP adaptable to patients’ individual needs, was believed to both improve process efficiency and quality of care, by doing everything right from the start.	
	Balancing resources and patient needs in the SRP: How to improve care quality and accessibility	Local staffing Designated time for SRP SRP competence Specialist support Available SRP interventions	Aiming for balance between available resources and workload, and between available SRP interventions and patient needs, was believed to prevent a negative spiral of suboptimal decision-making in individual cases resulting in more work, unfortunate outcomes, and increased patient suffering.	

This table describes the analysis structure and summarized results from the third of three qualitative analyses based on data collected with focus group interviews involving 31 professionals in the sick leave and rehabilitation process. Participants were from primary healthcare, the Swedish Social Insurance Agency, the Swedish Public Employment Service, and the Social Services. The analyses were intended to present decision support for managing quality driven change management within the SRP.

### The need for rules and regulations to enable coherent process governance

The participants stated the need for a shared SRP goal on a system level, process improvement work aiming for high-quality medical care, and meeting patients’ non-medical SRP needs. They also underlined the importance of consensus around clear definitions of central SRP concepts and responsibilities applicable in complex patient cases.

The participants described that the many different authorities in the SRP and the patients were working towards divergent goals: reaching better health, RTW (current or alternative), surviving economically, and independence from social benefits. The divergence of agendas and unclear division of responsibilities were perceived to result in professionals acting in opposition to each other, thereby bringing conflicting information to the patients.

Further, the participants perceived disagreements between agencies regarding the definition of work capacity. For instance, they mentioned that the Employment Agency assesses work capacity based on what an employer is willing to pay. In contrast, the Social Insurance Agency defines work capacity as ‘any activity capacity’. This difference was said to create difficulties in discerning who holds responsibility in various situations.

*"They’re playing a game of passing individuals around; that’s the problem. I feel sorry for these individuals being shuffled back and forth from the Employment Service to the Social Insurance Agency and Social Services; no one wants to take responsibility for them… it doesn’t work well." GP 14-4*

SRP regulations were designed to work best when diagnoses had well-defined symptoms and precise recovery times. However, the following issues were perceived to hinder effective collaboration in the patient’s best interest: 1) Elusive Symptoms: Symptoms that are difficult to identify or quantify. 2) Differing Definitions of Illness: Variations in how medical conditions are defined by different stakeholders. 3) Focus on Sick Leave: An assessment process that centers solely on determining entitlement to sick leave based on episodic symptom experience. 4) Focus on Working Capacity: An emphasis on regaining the ability to work, rather than ensuring a sustainable and supportive working environment. These factors collectively create challenges in achieving a collaborative approach that prioritizes the patient’s needs.

*”If we try to help them [the patients to regain work ability], and they receive financial compensation in collaboration with the Social Insurance Agency during that period… and we investigate their ability to perform any activity, which might not correspond to a job on the labour market…and then they are no longer entitled to sickness benefit because they have demonstrated that they can do something [even though they are not able to get a job]” Employment officer 27-1 from the Employment Agency*

The participants also described how the governance of healthcare and other SRP stakeholders had a two-sided focus on costs and savings over what is best for the patients. The perceived priorities were decided at a strategic level at each SRP stakeholder, aiming for local savings requirements without considering the implications for the whole system. Discontinuation of functions, formerly highly valued by stakeholders and patients, frequently led to very negative consequences for quality of care and SRP strain on other stakeholders, with a significant perceived impact on patient outcomes.

*"…so they closed down the local psychiatric clinic, and then those patients were just…shifted out to the primary healthcare centers […] a lot of patients who were essentially psychiatric patients!" Rehabilitation 22-4 - Coordinator role*

Narrow perspectives on process improvements were depicted to bring over-reliance on limited, short-sighted solutions needing a holistic perspective. As an example of the latter, even the role of the rehabilitation coordinator, which was seen as a resource that could bring about a positive solution, couldn’t compensate when the overall system failed.

The participants highlighted that stringent internal budget goals affect all stakeholders within the healthcare sector and other agencies. This was perceived to drive an SRP characterized by inflexibility and a ‘not-my-problem’ mentality, resulting in a never-ending workload without creating any additional value.

*"We have a sort of low-intensity war among all healthcare units trying to get everyone else to do the work. It would be interesting to know how much time, money, and energy are actually spent internally where you’re just… trying to fend off people […] it’s a budget war." Rehabilitation 16-5 - Coordinator role*

Economic interests were also described as influencing the role of the employers in SRP. While employer involvement was highlighted as a success factor, the participants found it problematic that a significant financial responsibility for occupational health and rehabilitation is placed on the patients’ employers, since employers simultaneously have the right to interpret what their responsibility entails. The participants perceived that many employers save money by downplaying the contribution of the working environment to ill health, and the need for occupational healthcare, and instead refer their employees to publicly funded primary healthcare.

*"In practice, it often happens that we somehow have to take on the case, even if we think it’s very clearly work-related. And then we end up being the last resort again…" GP 22-6*

### Challenges and opportunities in person-centered SRP: Professional collaboration, organizational priorities, and the need for enhanced leadership

The participants described that person-centered SRP care involved providing the right interventions at the right time by the right stakeholders and enabling professionals to perform their job effectively by providing them with favorable conditions. The participants expressed that person-centered organizational priorities meant making room for good professional judgment rather than standardized care processes, which was key to an SRP adaptable to patients’ needs. Using the expertise of different professions, on various levels of care, and from other agencies outside healthcare was described as a prerequisite for understanding and meeting patients’ individual SRP needs. In the focus groups, standardized approaches were mentioned to compensate for individual professionals’ shortcomings. However, it was also emphasized that the balance between medicalization and missed diagnoses was delicate in each particular SRP case, and that standards risked tipping the balance in either direction.

However, all participants identified room for improvement as their professional expertise was not being optimally used to work in a person-centered way in the SRP; 1) GPs were not allowed to acquire the overview needed to make a correct diagnosis, assess work capacity, and plan follow-up, 2) rehabilitating professions and rehabilitation coordinators were often involved so late that they found it challenging to make a difference, 3) psychologists were not used in the assessments to help understanding the patients’ resources as a basis for meeting the need for extra support, and 4) social insurance administrators at the Social insurance Agency, the Employment service, and social services received insufficient information from healthcare about patients’ abilities and limitations, which obstructed their planning of work-oriented interventions.

*"I think it would be great if the psychologist was involved in the whole rehabilitation process […] perhaps not always in treatment […] but as an assessment resource in the team […] so that you don’t make interventions that become ineffective due to for example personality structures in patients" Rehabilitation 14-1 - Psychologist profession*

The participants asserted that leadership in all agencies needed more understanding of each profession’s unique competencies. They also perceived a need for more knowledge among the professions regarding how various expertise could be utilized and what they might require to work in a person-centered manner. This was believed to complicate the decision-making process and priorities necessary to facilitate a person-centered SRP, both locally and centrally.

Further, the participants identified that consensus and coherent care in SRP requires an overall liability and trust. They saw great value in close collaboration among different professions, levels of care, and agencies within the SRP. At the same time, collaboration was considered challenging due to limited contacts and inadequate information transfer, making it difficult for each staff member to take responsibility.

*"I often find it difficult to know whether I can make demands, initiate some activity, or establish some planning for these individuals. Because we usually don’t get clear indications from healthcare [about how to proceed].” Social worker 27-3 from the Social Services*

The participants consistently called for more collaboration around individual patients. Collaboration was perceived as well-spent time that often contributed to problem-solving for the patient’s benefit through increased consensus. However, different participants had divergent views on the value of actual process-related tasks, usually performed by one actor and used by another. For example, GPs referred to medical certificates as an administrative burden, while social insurance administrators found well-written certificates helpful for adjusting work-related measures.

The participants agreed that healthcare plays a central role in providing the basis for understanding the causes of the patient’s suffering, and for planning coordinated care and rehabilitation efforts.

*"Often, the person has various health problems. They have pain issues, they have mental health issues […] Who is going to handle these issues? Should we start with the pain or should we start with the mental health issues? […] These questions are tough and if the primary healthcare center does not have its answer to this, my experience is that we get nowhere." Social worker 6-2 from the Social Services*

The importance of long-term responsibility for planning, revising, and driving the patient’s SRP over time was emphasized. The participants found it problematic that different GPs often get involved in the same SRP cases without having one doctor responsible for the patient. This was considered to lead to short-term rehabilitation plans, patients being uncertain about the next steps in the SRP, and delayed measures with the risk of worsening prognosis and prolonged sick leaves.

Continuity of therapeutical relations at all levels and for all professions in the SRP was considered to be a prerequisite for individually tailored care, enabling increased care quality and reduced costs in SRP. However, the participants identified that continuity should be sufficiently prioritized in the SRP, both within healthcare and other authorities. Participants described that a lack of knowledge about the patients was a barrier to involving patients in decisions about care, rehabilitation, and individualization of care.

*“We tend to .. talk about episodes, defined episodes, so .. healthcare is often measured by clear interventions or defined episodes ‘Psychotherapy starts here, we end psychotherapy at this date’, like that. But in reality, the person is still there, and the problem maintains over time [meaning sustainable health and workability may require longitudinal rather than episodic processes].” GP 13-3*

Further, the participants agreed that communication and collaboration could be facilitated through personal continuity and direct communication between involved professionals. They also highlighted that the patient’s process loses momentum and is prolonged when stakeholders at authorities and practitioners in healthcare are replaced.

*"Cases may require long-term planning, and then perhaps a person is needed to drive it; otherwise, it stalls" Social worker 27-6 from the Social Services*

### Balancing resources and patient needs in the SRP: How to improve care quality and accessibility

The participants advocated a more adequate balance between available resources - local staffing, designated time, competence, specialist support, SRP support and interventions - and patient SRP needs within healthcare and other agencies. Adequate resources were believed to prevent a negative spiral of suboptimal decision-making in individual patient cases, resulting in cascading of non-value-adding extra work, and poor patient outcomes.

Tight schedules were perceived to entail a focus shift from the patient’s best interest in SRP to managing the workload in daily practice. There needed to be more time at all levels in the SRP, and the participants described lack of time as leading to patients not being listened to, long periods of passive waiting, and a lack of follow-up. The participants felt they divided their time among as many patients as possible to maintain accessibility, which was described as leading to shortcuts. For example, an action that could be accomplished immediately was chosen over actions that required time but were perceived as the best for the individual patient.

*"The most obvious thing is about time, I think, and that we have a situation with waiting times and accessibility problems, and it makes you fall behind all the time […] ‘same day access’, in all stages would make us get to a plan faster, together with the patient." GP 13-3*

The participants argued that when compromises were made with the quality of care, it often resulted in both suffering for the patients and extra work for themselves. For example, lack of time led to difficulties in creating cohesive and individually tailored long-term SRP plans. Further, the participants described that it was frustrating if others needed more time to do their part with high quality, as it undermined the value of putting effort into their work for the patient.

*"I say one thing, write it in the journal, inform the doctor, and the doctor hasn’t had time to read it properly and suddenly says something else, and it becomes very confusing […] there should be time for cooperation from both sides" Rehabilitation 22-3 - Coordinator role*

Local variations in competence and organizational conditions were perceived to lead to unequal opportunities to adapt care in the SRP according to individual needs. For example, the participants described that their investment in their patients, competence or networks, local staffing with psychologists, doctors, and psychosocial teams, as well as local routines, including cross-professional collaboration, significantly impact on person-centeredness and coordination of SRP. Further, the SRP competence of GPs, which the participants considered very central to the outcome, varied greatly, and the lowest level needed improvement.

*"Some of them [doctors] can’t write certificates; they don’t understand what the Social Insurance Agency needs […] we have those who are lacking empathy […] doctors who handle things themselves, they don’t need any darn psychologists or rehab coordinators […] all these hired ones where people come to get sick-leave, with substandard certificates, no rehab plan […] conflict-avoidant, super nice, loved doctors who never think the patient is healthy […] and then we have skilled doctors, young doctors, super ambitious, long fine certificates, involve me directly, good at collaborating" Rehabilitation 16-2 - Coordinator role*

Access to a diverse range of specialized services, at the right time, was identified as an enabler of a person-centered SRP and RTW, through timely medical care and effectively tailored rehabilitation. However, the participants expressed that it was difficult to obtain specialist opinions for patients with vague symptoms, which was thought to be because specialist care could choose to limit their consultative assignments in case of work overload.

Also access to activities aimed at improving patients’ health and functional ability was considered far too low. For example, waiting times were often long for psychotherapy and work-oriented rehabilitation. In addition, support was often needed to manage a challenging everyday life, such as practical help with the care of children with special needs, to enable participation in rehabilitation. The participants argued that this complicated the SRP because high stress at home, like an increased workload, contributed to an imbalance between demands and abilities that could maintain ill health.

*"Then we end up in this… impossible situation where the problem is over there [what triggers or maintains ill health is an unsustainable life situation] and here we are in healthcare, and we can’t affect that because we have the patient here, and it’s something completely different over there. So… and that’s incredibly frustrating" Rehabilitation 16-4 - Coordinator role*

## Discussion

This study showed that the knowledge, willingness, and ambition to work in a person-centered and cohesive manner were present among all participants. Additionally, the result revealed that the previously identified gap between the desired outcome in SRP and the actual outcome [33, 34] seemed largely a consequence of the participants’ lack of understanding of how to meet patients’ needs under current conditions. Present rules and regulations, organizational priorities, and scarce resources were described as leading to impaired person-centeredness and evidence-based care, and quality losses within the SRP. Based on the result indicating that significant resources were allocated to managing patient flow, rather than fostering collaboration, we argue that the potential for quality improvements in SRP is substantial, in line with previous findings [1].

### Interpretation of results and comparison with previous work

From the theme *The need for rules and regulations to enable coherent process governance,* we found that the participants highlighted increased quality in SRP as an opportunity to save societal resources. This reasoning aligns with research showing the value of quality-driven organizational development [35, 40, 41]. However, the current study revealed that different professionals in SRP throughout the region shared the experience that improvement work is performed solely on a strategic level. Cost savings, rather than efficiency gains through increased quality, were perceived to drive improvement efforts in SRP, despite such prioritization being ineffective [35, 40–42]. We concluded that the depiction of SRP leadership that emerged in this study is far from the co-creating, and quality-driven, governance advocated in modern organizational research [35, 37–43].

The theme *Challenges and opportunities in person-centered SRP: Professional collaboration, organizational priorities, and the need for enhanced leadership* described that prioritizing collaboration in individual patient cases favors person-centeredness in SRP, by enabling consensus and benefiting from the combined specific expertise of each profession. Therefore, it is intriguing that previous intervention studies examining collaboration between the Social Insurance Agency and primary healthcare have yielded negative results [18–22]. The value of cooperation, without further specifying how, could hence be disputed and, interestingly, we haven’t found any guidelines concretizing how to enable effective collaboration in clinical practice. However, based on the current study and its companion studies [33, 34], we argue that the adverse outcomes in SRP collaboration studies could partly be attributed to SRP’s strict diagnostic perspective - potentially leading to long-term neglect of non-medical factors that contribute to illness, and to suboptimal use of each profession’s unique expertise, thus prolonging sick leave. This reasoning aligns with the bio-psycho-social model, which emphasizes the significance of social and psychological factors in medical conditions, particularly in chronic and diffuse cases [51]. Juxtaposing our results and previous research, we concluded that enabling hands-on collaboration in individual cases is central to the outcomes of SRP. Still, successful outcomes presuppose that non-medical factors are considered within the SRP, relationship continuity, and that each profession’s expertise is optimally used in SRP [18–22, 33, 34].

The importance of relationship continuity in enabling high-quality processes and person-centeredness was emphasized as a second organizational priority favoring person-centeredness in SRP, adding a new perspective to the guidelines for person-centered care [31]. The current result complements previous SRP guidelines and research about the value of relationship continuity for specific professions [23–25, 29, 30], by highlighting the importance of relationship continuity as a general principle for all professionals involved in the multi-disciplinary SRP teamwork, within healthcare and other agencies.

Minimizing unwarranted practice variations, while also allowing room for professionals to exercise judgment for the long-term sustainable health and work capacity of patients, was pointed out to be a third organizational priority favoring person-centered care. We interpreted that adopting standardized methods for collaboration to make use of the expertise of different professionals can enhance the quality of SRP care, but standardizing the care content could degrade process quality.

The theme *Balancing resources and patient needs in the SRP: How to improve care quality and accessibility*, showed that the participants called for adequate working conditions in SRP, and underlined the need for practical medical information about the limitations of the disease, in line with previous reports [6, 7]. The participants further argued about the importance of sufficient time for listening to the patient, and of applying a person-centered approach, which aligns with the Swedish standard for person-centered care [31]. These findings are consistent with organizational research emphasizing the value of high-quality care to avoid mistakes [35, 36], and the necessity for adequate resources to enable change [43].

The result also identified knowledge gaps, relevant to practicing a person-centered SRP, at multiple societal levels. Arguing knowledge gaps at various societal levels differed from the standard of person-centered care, which emphasizes the importance of healthcare professionals being trained to explore patients’ narratives [31]. Based on our findings and in line with implementation principles [43], we concluded that insufficient SRP knowledge at multiple societal levels could be a barrier to form effective laws, regulations, and guidelines to create a person-centered SRP with high quality.

### Considerations for a purposeful sick leave and rehabilitation process

Achieving a more efficient SRP requires holistic improvement efforts. We concluded that involving all professions in improvement work, at different organizational levels and across administrative boundaries, is a key issue to capture different professional perspectives. Similar conclusions were drawn in previous research [37–39].

However, we observed tensions between different healthcare professions during the focus groups. We interpreted these tensions as partly rooted in historical power structures and reactions to them, as well as a result of representatives from different professions sometimes missing the perspectives of others. However, we found that exploring what different professions perceived to influence the outcomes in SRP, how they can contribute to better care in SRP, and the necessary prerequisites to make use of their specific expertise - together with a ”how do we meet this challenge together”-perspective - contributed to a fruitful dialogue in this study. We concluded that bringing together various professions around a common goal of enabling quality care, shifts the focus to person-centered care and highlights the potential contributions of different areas of expertise, while avoiding the "not-my-table" mentality.

The potentially significant socioeconomic benefit of an optimized SRP is reduced sick leave. However, while SRP work is conducted at the local and regional levels, the resulting economic benefits are realized in a different sector of the economy. In line with previous organizational research [35, 36], our findings suggest that it might be a cost-effective strategy in the long run for society to redistribute resources to enable prioritizing adequate organizational conditions favoring person-centeredness, and balance between resources and assignments to allow timely interventions in SRP – both proper healthcare and societal interventions for ill-being that is not medical. Further, based on this study, and consistent with implementation research [43], we also argue that increased SRP knowledge, organizational prerequisites fostering person-centeredness, and supportive leadership enabling coherent rules, regulations, and process governance is necessary to transition to a person-centered SRP.

### Strengths and limitations

This study put organizational knowledge into practice when turning to SRP professionals for input on SRP improvement work, asking what they need to help their SRP patients. The selected qualitative study design and data collection through focus group interviews proved well-suited for the study’s objectives. The dialogues within the focus groups were straightforward and characterized by a wealth of detailed personal experiences and contextual examples, which enabled the researchers to grasp the intricate nature of the SRP.

Although the participants worked in different locations and public service organizations, their views on regional SRP governance aligned, and they suggested similar SRP improvements. We want to emphasize the striking consistency in the narratives that emerged in this study.

The interdisciplinary research approach was a key strength of the study as analyzing the data through various research perspectives challenged our initial focus on a single research field, allowing for a broader, more in-depth understanding. Additionally, having to explain each perspective at a fundamental level sharpened the analysis. Nevertheless, different research paradigms aim to address distinct questions. Separate sets of themes and subcodes were used to accommodate these differences.

However, qualitative research methods are designed to help reveal a target group’s feelings, beliefs, and experiences about a specific topic and to understand their behaviors. Qualitative findings do not prove anything as the selection of participants is strategic, not random, and the findings are based on statements of a few people. Carefully used, descriptive findings from qualitative research are crucial for making good context-related decisions and planning quantitative studies.

The findings of this study, given the qualitative methodology, are not intended to serve as a definitive demonstration of the factors affecting SRP. Nevertheless, our results identified several prerequisites for successful SRP management in Västra Götaland, Sweden, that coincided with previous findings: 1) critical factors defining proficient healthcare teamwork to solve complex tasks include contextual consensus, mutual dependence and coordination, team members’ collective expertise matching the task, clearly defined responsibilities, and well-defined leadership. Similar results about contextual consensus and mutual dependence were found previously [52–54], 2) Relationship continuity being a key factor for proficient primary healthcare. The importance of a continuous relationship between healthcare professionals and patients was highlighted in our as well as in previous research [28, 55], and 3) That Adequate conditions (such as time and other resources) was a prerequisite for implementation success was found in the present article, and was also stressed by The Swedish National Board of Social Affairs and Health [43]. We concluded that our qualitative findings may partially apply to multi-professional problem-solving of SRP and other complex tasks in primary healthcare.

## Conclusion

The results highlighted the need for clarified rules and regulations in SRP, person-centered organizational priorities, and resources aligned with the mission, to enable efficient, individualized support to medical and non-medical problem-solving. The findings offered qualitative guidance in enabling SRP person-centeredness and efficiency. To allow cautious collaboration between professionals, creating synergy effects from combining their expertise, and avoiding ”entrapment effects” in SRP, primary healthcare centers, rehabilitation units and agencies must prioritize collaboration, overall liability, and continuity of relations at all levels. As a next step, the findings could be translated into a set of questions for a larger group of professionals to answer, allowing to either confirm or challenge our findings. Finally, further research is needed to explore why managers and decision-makers, on organizational meso- and macro levels, make decisions that are perceived to counteract person-centeredness and efficiency at the micro-level of SRP.

## Supplementary Material

Supplementary_information_The_Swedish_sick_leave_and_rehabilitation_process_240510 (1).docx

S1 Interview guide.docx

## Data Availability

Complete interview data cannot be made publicly available for ethical and legal reasons according to the Swedish regulations of the “Act concerning the Ethical Review of Research Involving Humans (2006:460)” (https://www.kliniskastudier.se/english/for-researchers/laws-regulations/act-concerning-ethical-review-research-involving-humans--.html) and the Swedish Ethical Reviews Authority (https://etikprovningsmyndigheten.se) Data are however available from the corresponding author on reasonable request.
