# Supplementary material for: A systems perspective on gaps in the person-centered sick leave and rehabilitation process: a Swedish interview study
Source: Scand J Prim Health Care. 2024 Dec 2;43(2):324–37. doi: 10.1080/02813432.2024.2434123 (PMC12090277; doi:10.1080/02813432.2024.2434123)
Supplement: Supplementary_information_The_Swedish_sick_leave_and_rehabilitation_process_240510 (1).docx [file IPRI_A_2434123_SM8664.docx]

**Supplementary information 1: The Swedish sick leave and rehabilitation process**Swedish healthcare is divided into 21 self-governing regions, and publicly funded. In the Västra Götaland Region, one of the largest regions, the staffing at the primary healthcare centers includes general practitioners (GPs), district nurses, psychologists, psychotherapists, and rehabilitation coordinators [1]. Rehabilitation coordinators have an in-depth understanding of the SRP and a degree in either health science, social work, or psychology. They are tasked with being the patient's

contact in SRP and coordinating SRP communication between process actors [2]. Primary healthcare also includes rehabilitation units, with physiotherapists and occupational therapists, where patients can turn without a referral. Rehabilitation units may or may not be co-located with a healthcare center [3].

Sick leave certificates are issued in primary healthcare by GPs if the illness is not exclusively work-related, or requires specialist care. The certificates are based on assessing diagnosis, functional- and activity impairments, and workability about work requirements [4]. The employer pays for the first two weeks of sick leave. After that, the Social Insurance Agency is responsible for assessing the right to sick leave based on the medical certificate, coordinating vocational rehabilitation, and calling reconciliation meetings when needed [4]. The employer remains responsible for making work adjustments that facilitate the employee to return to work (RTW) and for engaging occupational health services when needed [5].

The Employment Agency is responsible for SRP coordination, and planning work-oriented rehabilitation for job-seeking patients with health-related impaired work ability entitled to unemployment benefits [6]. Similarly, the Social Services are responsible for financial aid and SRP management for patients who are neither entitled to sickness nor unemployment benefits [7]. The Employment Agency, and the Social Services, plan work-oriented rehabilitation based on medical statements from healthcare describing patients’ health-related resources and restrictions.

The responsibility for coordinating the patient’s SRP passes from the Social Insurance Agency to the Employment Agency (or the Social Services) when patients have impaired workability in their current employment and preserved workability in another job, usually after 180 days [8]. When needed, the Social Insurance Agency, the Employment Agency, the Social Services, and the regions may jointly finance local work-oriented rehabilitation interventions and collaborate in individual SRP cases [9].

**References**

1. Region Västra Götaland. Krav- och kvalitetsbok Vårdval Vårdcentral (Requirements and quality book Choice of care Primary care center). Gothenburg: Region Västra Götaland; 2022.
2. Swedish Association of Local Authorities and Regions. Koordineringsinsatser försäkringsmedicin (Coordination insurance medicine) [Internet]. Stockholm: Swedish Association of Local Authorities and Regions;2023 [Updated 2024-01-26; cited 2024-04-09]. Available from <https://skr.se/skr/halsasjukvard/forsakringsmedicin/koordineringsinsatser.1033.html>
3. Region Västra Götaland. Krav- och kvalitetsbok Vårdval Rehab (Requirements and quality book Choice of care Rehabilitation) [Internet]. Gothenburg: Region Västra Götaland; 2022. [Cited 2024-04-09]. Available from [https://mellanarkiv-offentlig.vgregion.se/alfresco/s/archive/stream/public/v1/source/available/sofia/osn12568-2439792-2/native/Krav-%20och%20kvalitetsbok%20Vårdval%20Rehab_2023_%20beslutad%202022-09-28.pdf](https://mellanarkiv-offentlig.vgregion.se/alfresco/s/archive/stream/public/v1/source/available/sofia/osn12568-2439792-2/native/Krav-%25252520och%25252520kvalitetsbok%25252520V%2525C3%2525A5rdval%25252520Rehab_2023_%25252520beslutad%252525202022-09-28.pdf)
4. The Swedish Social Insurance Agency. Intyg för sjukpenning (Certificate for sickness benefit) [Internet]. Stockholm: The Swedish Social Insurance Agency; 2023 [Updated 2024-03-04]; cited 2024-04-09]. Available from: <https://www.forsakringskassan.se/halso-och-sjukvarden/sjukdom-och-skada/intyg-for-sjukpenning>
5. Systematiskt arbetsmiljöarbete Arbetsmiljöverkets föreskrifter om systematiskt arbetsmiljöarbete och allmänna råd om tillämpningen av föreskrifterna (Systematic work environment work The Swedish Work Environment Authority's regulations on systematic work environment work and general advice on the application of the regulations) (AFS 2001:1) [Internet]. Stockholm: The Work Environment Agency. [Cited 2024-04-09]. Available from: <https://www.av.se/globalassets/filer/publikationer/foreskrifter/systematiskt-arbetsmiljoarbete-foreskrifter-afs2001-1.pdf>
6. Förordning om den arbetsmarknadspolitiska verksamheten (Ordinance on labor market policy activities) (SFS 2000:628). Stockholm: Ministry of Labour. [Cited 2024-04-09]. Available from: <https://www.riksdagen.se/sv/dokument-och-lagar/dokument/svensk-forfattningssamling/forordning-2000628-om-den_sfs-2000-628/>
7. Socialtjänstlagen (The Social Services Act) (SFS 2001:435). Stockholm: Ministry of Social Affairs. [Cited 2024-04-09]. Available from: <https://www.riksdagen.se/sv/dokument-och-lagar/dokument/svensk-forfattningssamling/socialtjanstlag-2001453_sfs-2001-453/>
8. The Swedish Social Insurance Agency. Rehabiliteringskedjan (The rehabilitation flowchart) [Internet]. Stockholm: The Swedish Social Insurance Agency; 2023. [Updated 2024-03-13]; cited 2024-04-09]. Available from: <https://www.forsakringskassan.se/om-forsakringskassan/vart-uppdrag/om-socialforsakringen/rehabiliteringskedjan>
9. Lag om finansiell samordning av rehabiliteringsinsatser (Act on financial coordination of rehabilitation efforts) (SFS 2003:1210). Stockholm: Ministry of Social Affairs. [Cited 2024-04-09]. Available from: <https://www.riksdagen.se/sv/dokument-och-lagar/dokument/svensk-forfattningssamling/lag-20031210-om-finansiell-samordning-av_sfs-2003-1210/>
